# Dairy farming and climate parameters: an analysis of high productivity milk-producing cities in Minas Gerais, the leading dairy state in Brazil

**DOI:** 10.29374/2527-2179.bjvm008525

**Published:** 2025-12-16

**Authors:** Marcus Vinícius Dias-Souza, Gustavo Augusto Bitancourt Oliveira, Amanda de Barros Martins

**Affiliations:** 1 Escola de Educação e Saúde, Centro Universitário Católica do Leste de Minas Gerais, Coronel Fabriciano, MG, Brazil.

**Keywords:** dairy cattle, climate change, milk production, dairy farming, gado leiteiro, mudanças climáticas, produção de leite, pecuária leiteira

## Abstract

Brazil is the world’s fifth largest milk producer, and Minas Gerais is the leading milk-producing state. Most of the state production comes from small and medium-sized family farms, and only a small number of cities have high productivity metrics. The current climate change scenario poses technical challenges to milk production, affecting the health and the productivity of the cattle. Here we evaluated dairy farming in Minas Gerais state, considering productivity and official climatic data. We analyzed official data from different government databases, and among the 853 cities, 12 were classified as high-production cities (>80,000 L/year), of which six had complete climatic records. Pompéu emerged as the top producer with high precipitation rates, whereas Patos de Minas showed the highest precipitation, with irregular distribution. Using quadratic polynomial regression, we found that precipitation significantly influenced production (R^2^=0.8993, p=0.0076). Temperature alone had a negligible effect (R^2^=0.1995, p=0.7174). Principal Component Analysis identified distinct climatic patterns among the cities, with January and December being the wettest periods. Notably, high-productivity areas maintained moderate temperatures (21–23°C) and lower animal densities (km^2^). Our data open doors for further investigation into the interplay between climatic changes and zootechnical parameters of interest in milk production.

## Resumo

O Brasil é o quinto maior produtor mundial de leite, e Minas Gerais é o principal estado produtor de leite. A maior parte da produção do estado vem de pequenas e médias propriedades familiares, e são poucas as cidades que tem índices de alta produtividade. O cenário atual de mudanças climáticas impõe desafios técnicos para a produção de leite, impactando a saúde e a produtividade do gado. O objetivo deste estudo foi avaliar a produção leiteira no estado de Minas Gerais, considerando a produtividade e dados climáticos oficiais. Foram analisados dados de diferentes fontes oficiais governamentais, e entre as 853 cidades do estado, 12 foram classificadas como cidades de alta produção (> 80.000 L/ano), das quais seis tinham registros completos de dados climáticos. A cidade de Pompéu apresentou os maiores índices de produtividade entre as cidades com maior índice de precipitação, enquanto Patos de Minas apresentou o maior índice de precipitação entre as cidades de alta produtividade, apesar da distribuição irregular ao longo do ano. Usando regressão polinomial quadrática, constatou-se que a precipitação influenciou significativamente a produção leiteira (R^2^ = 0,8993, p = 0,0076). A temperatura, isoladamente, não teve efeito significativo (R^2^ = 0,1995, p = 0,7174). Por meio da Análise de Componentes Principais, foram identificados padrões climáticos distintos entre as cidades, sendo os meses de janeiro e dezembro os mais chuvosos. As áreas de alta produtividade mantiveram temperaturas moderadas (21–23 °C) e menor densidade animal/km^2^ no período estudado. Os dados deste estudo apontam para a necessidade de investigações mais aprofundadas sobre a interação entre mudanças climáticas e parâmetros zootécnicos de interesse para a produção de leite.

## Introduction

Milk is a highly nutritious food that provides proteins, vitamins (such as A, D and B_12_), and minerals such as calcium and phosphorus. It is also essential for the production of widely consumed products such as cheese, yogurt, and butter (Pereira, 2014). Dairy cattle farming is a vital aspect of the dairy sector that directly influences the quality and yield of milk. Dairy producers strive to improve cattle genetics to enhance productivity, which can affect the health and welfare of animals (Cole & Silva, 2016). Dairy cattle are more susceptible to health issues such as mastitis, laminitis and metabolic disorders than non-dairy cattle (Steeneveld et al., 2024). Therefore, dairy farming must adhere to technical standards that ensure animal welfare without compromising commercial objectives (Minas Gerais, 2019; Sousa et al., 2023). These challenges are even more complex owing to the increased climate alterations observed in Brazil and worldwide, especially in the past two decades.

Milk production is one of the main economic activities in Brazilian agribusiness, with Brazil currently ranking among the five largest milk producers worldwide (Telles et al., 2020). Since the 1990s, the state of Minas Gerais (MG) has been the national leader in milk production, an activity deeply rooted in the state’s culture since the 19^th^ century (Osei-Amponsah et al., 2020; Ribeiro et al., 2024). This tradition, combined with technological advancements and considerable land availability, has fostered a solid and diversified production chain, ranging from small and medium-sized family farms to highly technified dairy operations (Martins et al., 2003; Pereira et al., 2016). Many producers in the state employ strategies such as cattle genetic improvement and milking automation, increasing per-animal output and boosting the dairy sector (Costa et al., 2025; Cunha et al., 2018; Ferrazza & Castellan, 2021). Nevertheless, managing cattle under adverse climate conditions remains a challenge, regardless of the farming system (Scoones, 2023).

The most widespread production system in Brazil for dairy and beef cattle is extensive pasture-based farming (Ferrazza & Castellan, 2021; Gomes et al., 2018). Approximately 30% of the total milk production in Brazil is from MG, and nearly 70% of this production comes from small- and medium-sized family farms, often organized as working cooperatives to effectively participate in dairy chain supplies of both the public and private sectors (Agência Minas, 2024; Minas Gerais, 2019). In this context, climate challenges linked to global warming, such as heatwaves, prolonged droughts, river overflows, and floods that devastate farm properties (and urban areas), can significantly affect dairy cattle health and productivity (Ferrazza & Castellan, 2021; Ribeiro et al., 2024). High temperatures may lead to heat stress, reduced feed intake, and lower milk yield, whereas droughts can limit access to quality forage and water (Dalcin et al., 2016; Sammad et al., 2020). Breed selection and resilient infrastructure are both critical for minimizing climate-related impacts on welfare and productivity (Sammad et al., 2020, Scoones, 2023; Steeneveld et al., 2024).

Studies on milk production metrics in Brazil and its states are crucial for technical development of the sector (Martins et al., 2003). As one of the world’s largest milk producers, with MG as the leading state, understanding this activity’s dynamics concerning climate vartions allows realistic assessments, improvements on the efficiency of processes, and also open doors for innovations that meet technical, economic, and environmental demands in tropical livestock farming. Nevertheless, the collection, analysis, and interpretation of data faces obstacles, particularly because official records often outdated, dispersed, and poorly traceable. Given the exposed, this study aims to analyze and map high-productivity dairy farming in Minas Gerais, considering official climatic parameters from the cities that meet the technical criteria of this category of milk production.

## Materials and methods

### Data collection

Data on milk production in Minas Gerais for the year 2022 were obtained from the Milk Intelligence Center (*Centro de Inteligência de Leite - CILeite*) of the Brazilian Agricultural Research Corporation (*Empresa Brasileira de Pesquisa Agropecuária - EMBRAPA*). The year was selected as the most recent post COVID-19 pandemic period available on the platform. Due to restrictions on human mobility and economic activities during the pandemic, data from 2020 were not considered for analysis.

The CILeite/EMBRAPA platform generated maps with distinct parameters for each municipality in Minas Gerais, including milk production (thousand liters/year), production density (L/km^2^), cow density (number of animals/km^2^), total milked cows (number of animals/year), and animal productivity (volume of milk produced per animal per year). Although monthly milk production values per city were not publicly available at the time of writing, annual production volumes were retrieved from the Brazilian Institute of Geography and Statistics (*Instituto Brasileiro de Geografia e Estatística* - IBGE) database.

The climatic data were obtained from the National Institute of Meteorology (*Instituto Nacional de Meteorologia - INMET*) via its database. The selected parameters were precipitation levels and dry-bulb air temperature, recorded hourly in a daily basis. Only high-production municipalities (annual output >80,000 liters, defined by CILeite/EMBRAPA) were included. This study used open-access public data; thus, no ethics committee approval was required.

### Data processing

CILeite/EMBRAPA cartographic data were integrated into Google Earth Engine using JavaScript scripts developed with ChatGPT AI to identify cities with milk production ≥80,000 liters/year. The results were manually cross-checked with official state government maps. Publicly available farming system data were extracted from the official municipal websites. For climatic variables, monthly means and standard deviations were calculated to estimate the annual averages of temperature and precipitation per city.

### Statistical analysis

Climatic data normality and homoscedasticity were assessed using Shapiro-Wilk and Bartlett’s tests, respectively. Yeo-Johnson transformation was applied to approximate the data to normal distribution. Temperature and precipitation differences were evaluated using ANOVA and post-hoc Tukey’s test. Pearson’s correlation was used to test production-climate relationships, followed by multiple linear and quadratic polynomial regression to assess variable effects on milk yield. These analyses were carried out in Bioestat 5.0 for Windows. Principal Component Analysis (PCA) was performed in PAST 5.2 for Windows to reduce climatic data dimensionality and identify intercity similarity patterns. Significance was set at p<0.05.

## Results

### Identification of high productivity cities

**Figure 1:**
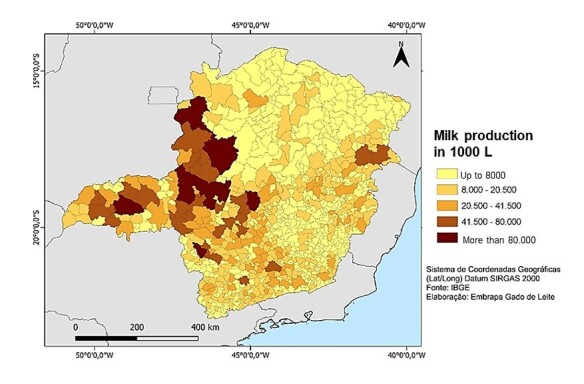
Minas Gerais cities classified by color scheme according to milk production capacity. Retrieved from CILeite/EMBRAPA platform. The image legends were manually translated to english.

**Figure 2:**
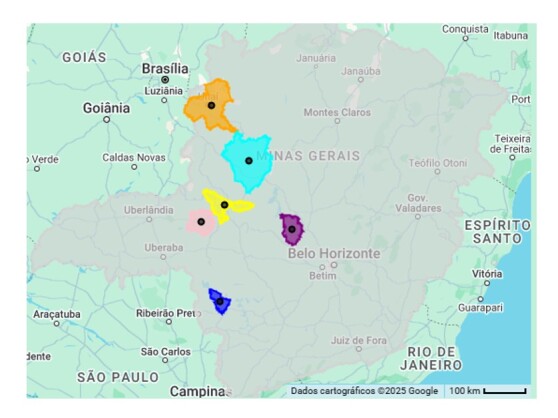
Location of cities with available official climatic data: Passos (dark blue), Patos de Minas (yellow), João Pinheiro (light blue), Unaí (beige), Patrocínio (pink) and Pompeu (purple), at Minas Gerais state (gray). Prepared with Google Earth Engine.

Twelve cities with milk production exceeding 80,000 liters/year were identified in five planning regions established by the state of Minas Gerais (Figure 1), namely: Pompéu (Central region), Passos (Southern region), Prata (Triângulo Mineiro), Unaí, João Pinheiro (Northwest region), Coromandel, Patrocínio, Patos de Minas, Lagoa Formosa, Carmo do Paranaíba, Rio Paranaíba and Tiros (all from Alto Paranaíba region). Among these, only six cities had available climate data (Figure 2).

### Comparative analysis of CILeite/EMBRAPA data

**Figure 3:**
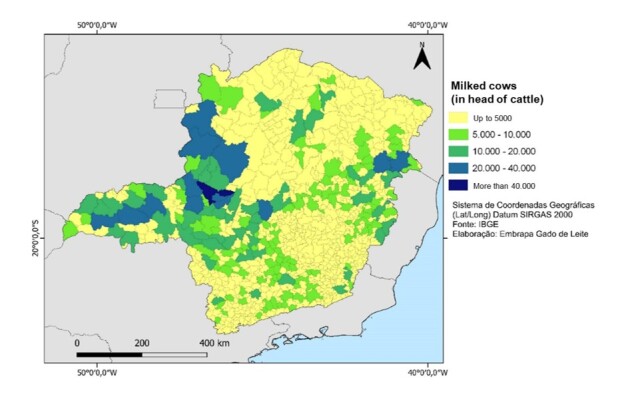
Cities according to total number of milked cows. Prepared with public data from CILeite/EMBRAPA platform. The image legends were manually translated to english.

**Figure 4:**
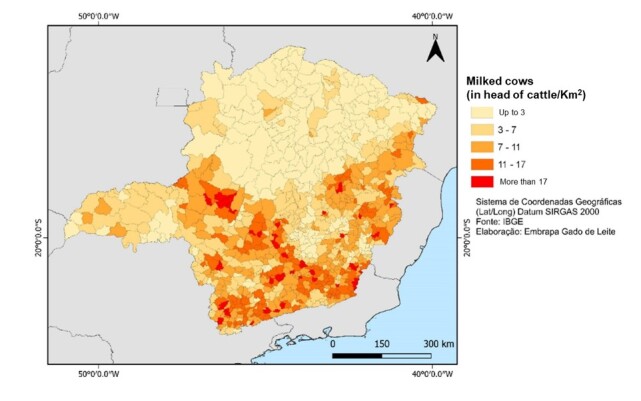
Total milked cows per Km^2^ in Minas Gerais cities. Prepared with data from CILeite/EMBRAPA platform. The image legends were manually translated to english.

**Figure 5:**
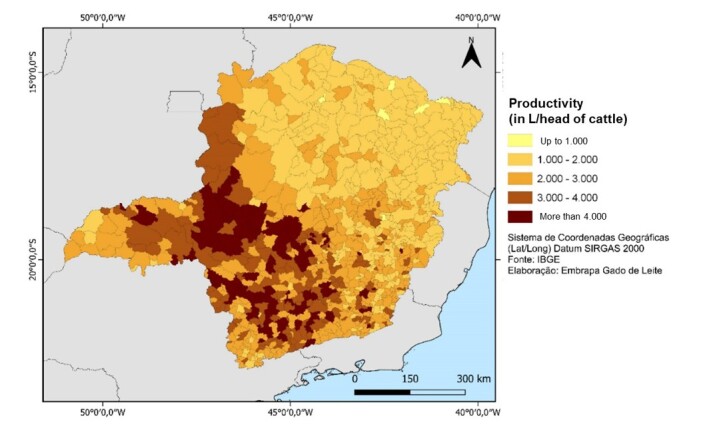
Average milk volume produced per animal in Minas Gerais cities. Source: prepared with data from CILeite/EMBRAPA platform.

CILeite data indicated that the 12 cities with high productivity had elevated numbers of milked cows. Pompéu stands out as the only city with more than 40.000 milked animals in 2022 (Figure 3). The geographic distribution of milked cows per km2 showed a dispersed pattern in the 12 high-production cities (Figure 4), whereas a higher number of animals per km^2^ was observed in cities with fewer milked animals per year (Figure 3) and lower milk production (Figure 1). In some cities with a higher number of animals per km^2^, producers have achieved over 4000 liters of milk per cow per year (Figure 5). However, the total production did not exceed 80 thousand liters annually, excluding those from the high-production city group. Among the 12 evaluated cities, João Pinheiro stands out for its dispersed cow distribution, intermediate milk yield per animal, high number of milked animals, and total productivity exceeding 80 thousand liters of milk annually (Figures 1 to 4).

### Dairy cattle farming systems and climatic data analysis

**Figure 7:**
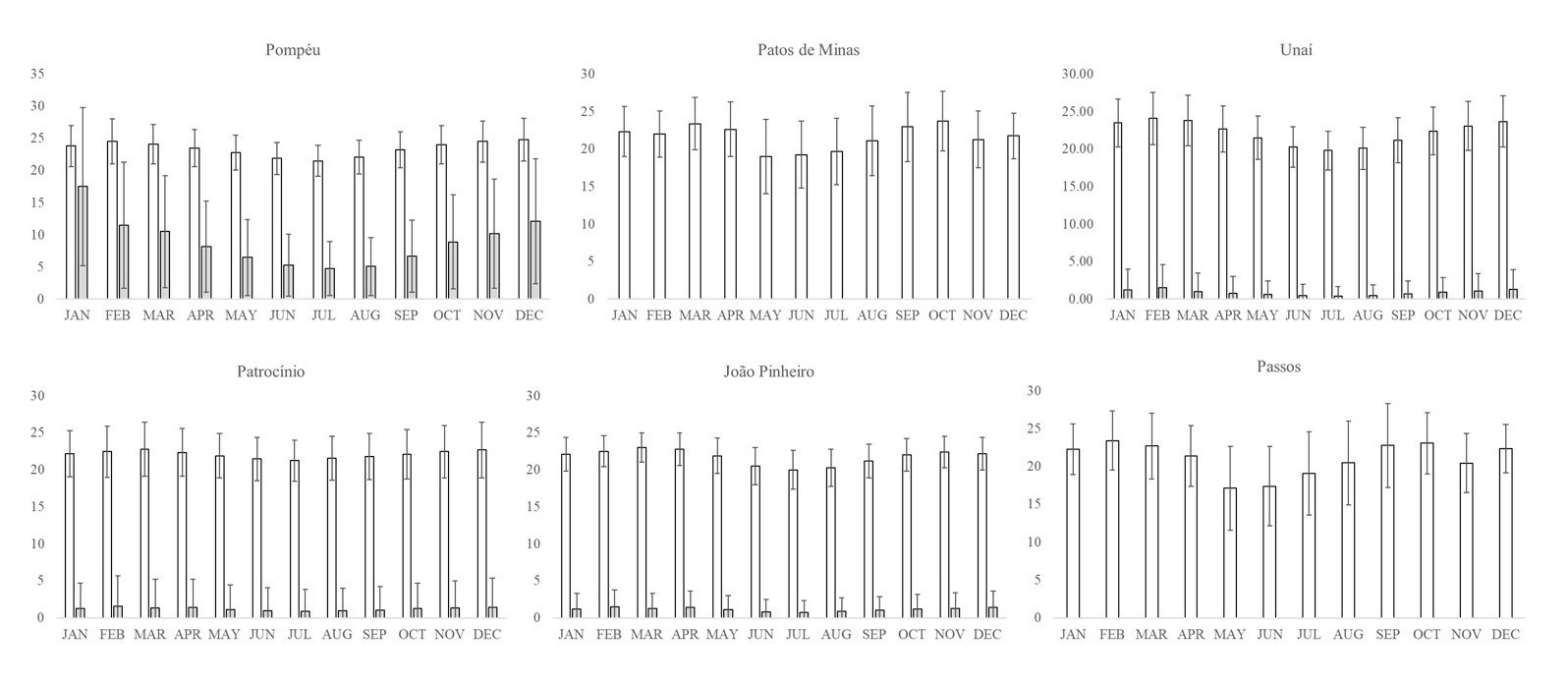
Temperature averages (white color, in °C) and precipitation (gray color, in mm of rain per day) for the cities of Pompéu, Patos de Minas, Unaí, Patrocínio, João Pinheiro, and Passos. Precipitation data were not included for the remaining cities due to the high number of months without precipitation, resulting in annual averages below 0.1 mm/day.

Among the evaluated cities, only Patrocínio, Patos de Minas, Pompéu, Unaí, João Pinheiro and Passos had official 2022 climatic data available (Figure 2). The metrics related to these data are represented in Figure 7. In these locations, the dairy cattle farming system is essentially intensive, with the Girolando breed being the most common (with different genetic fractions of Gir and Holstein breeds), followed by the Holstein breed. There are no available data about the most used genetic fractions.

The climatic pattern of Passos was marked by low precipitation: the annual average was only 0.0311 mm/day (total of 330.58 mm/year), with December and February being the rainiest months. From May to August there was a long dry period. The average annual temperature was 21°C, with the hottest months being October (23.06°C) and February (23.4°C), while May (17.11°C) and June (17.39°C) were the coldest.

The city of Unaí showed an average annual precipitation of 0.9 mm/day (1211.6 mm for the whole year), with rains concentrated mainly in the first quarter of the year. Between the months of January and March, 300 mm of precipitation accumulated, with January being the rainiest month. The average annual temperature was 22.1°C, with February (24.1°C) and December (23.7°C) being the hottest months. July (19.8°C) and June (20.3°C) were the coldest months.

At the city of Pompeu there was high daily precipitation volume (8.7 mm/day, 1250.8 mm for the whole year), with non-linear distribution throughout the months: January was the rainiest month, but there was drought from June to August. The average annual temperature was 23.5°C, with December (24.8°C) and November (24.5°C) being the hottest months, while July (21.5°C) and June (21.9°C) were the mildest.

The city of João Pinheiro showed an average annual temperature of 21.8°C, with January being the rainiest month. The hottest months were March (23.0°C) and February (22.5°C), while July (20°C) and June (20.5°C) recorded the lowest averages. Only 25 days in the entire year had recorded precipitation, being the shortest rainy period among the evaluated cities.

The city of Patrocínio showed annual average temperatures of 22.12°C and average annual precipitation of 1.23 mm/day (1638.4 mm for the whole year), with February being the rainiest month. Temperature varied moderately throughout the months, with the highest values in December (22.67°C) and March (22.78°C), while July (21.23°C) and June (21.45°C) were the coldest. This was the city with the longest rainy period among those investigated, with a total of 85 days with precipitation.

The city of Patos de Minas had an average annual temperature of 21.6°C and the highest total annual precipitation among the evaluated cities (1940.6 mm), with January being the rainiest month. However, precipitation frequency was irregular throughout the year, to the point that the daily precipitation average exceeded 0.5 mm/day only in December, January and February, with no precipitation registered from May to September. The months of October (23.76°C) and March (23.37°C) were the hottest, while May (19°C) and June (19.26°C) were the coldest.

February and March stood out as the hottest months among these cities, while June and July were generally the coldest. From a statistical perspective, the city of Pompéu showed higher temperatures throughout the year than Passos (p<0.01) and Patos de Minas (p<0.05). There were no other significant differences among the analyzed cities.

Considering total precipitation throughout the year, we only detected statistically significant difference between the volumes recorded for Pompéu and Passos (p<0.05). Taken together, the analysis of monthly total volumes indicates that, regardless of the city, the first four months of the year and the months of October and December are the periods with highest precipitation frequency (Table 1). No significant differences were observed in other comparisons between recorded monthly frequencies.

**Table 1:** Results of statistical analyses on the average monthly precipitation volume in the cities.

**Compared months**	**p-value**	**Best-performing month**
JAN–JUN	0.007868*	JAN
JAN–JUL	0.02656	JAN
FEB–MAY	0.03766	FEB
FEB–JUN	0.002498*	FEB
FEB–JUL	0.009222*	FEB
MAR–MAY	0.03682	MAR
MAR–JUN	0.002431*	MAR
MAR–JUL	0.00899*	MAR
APR–JUN	0.03204	APR
JUN–OCT	0.007164*	OCT
JUN–DEC	0.009346*	DEC
JUL–OCT	0.02438	OCT
JUL–DEC	0.03106	DEC

JAN: January; FEB: February; MAR: March; APR: April; MAY: May; JUN: June; JUL: July; OCT: October; DEC: December;

* Highly significant values (p < 0.01).

The multiple linear regression, although not significant (p = 0.184), showed a moderate coefficient of determination (R^2^ = 0.6768; adjusted R^2^ = 0.4923), suggesting that approximately 67% of production is influenced by climatic parameters, with a stronger statistical trend for precipitation (t = 2.4961, p = 0.0880) than for temperature (t = -0.5088, p = 0.6460). For each 1 mm increase in precipitation, an increase of 73.08 liters in milk production is estimated in a scenario of stable temperature (b1 = 73.0814). Moreover, for each one-degree increase in average temperature, an annual decrease of more than 10,000 liters of milk was estimated in a scenario of stable precipitation (b2 = -10202.7619). Similarly, Pearson's test suggested correlation between milk production and precipitation (R^2^ = 0.6490, p = 0.0530), which was not detected for temperature (R^2^ = 0.0626, p = 0.6324).

**Figure 6:**
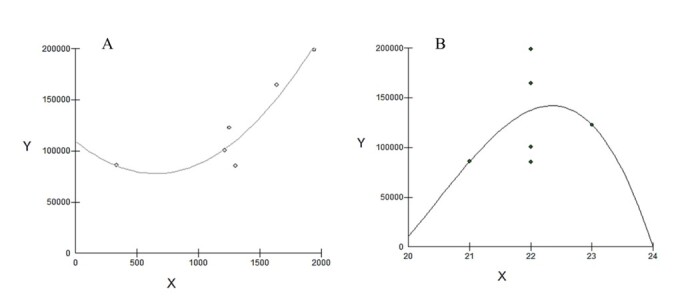
Quadratic polynomial regression for milk production and precipitation (A) and temperature (B). The X-axis represents precipitation in both graphs.

Given these findings, annual milk production data, precipitation and average annual temperature were evaluated by quadratic polynomial regression (Figures 6A and 6B). The model was statistically significant for precipitation (p = 0.0076) and explained about 83% of the variation in milk production (R^2^ = 0.8993; adjusted R^2^ = 0.8321) in a nonlinear relationship. However, for temperature, the regression showed no statistical significance, and low effect (R^2^ = 0.1995; adjusted R^2^ = -0.3342; p = 0.7174).

**Figure 8:**
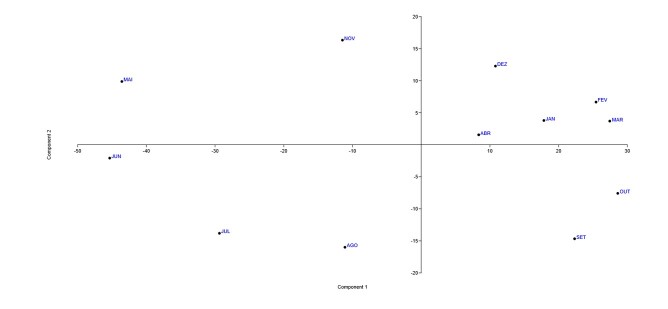
PCA results for months of the year regarding temperature, taken all the cities together.

**Figure 9:**
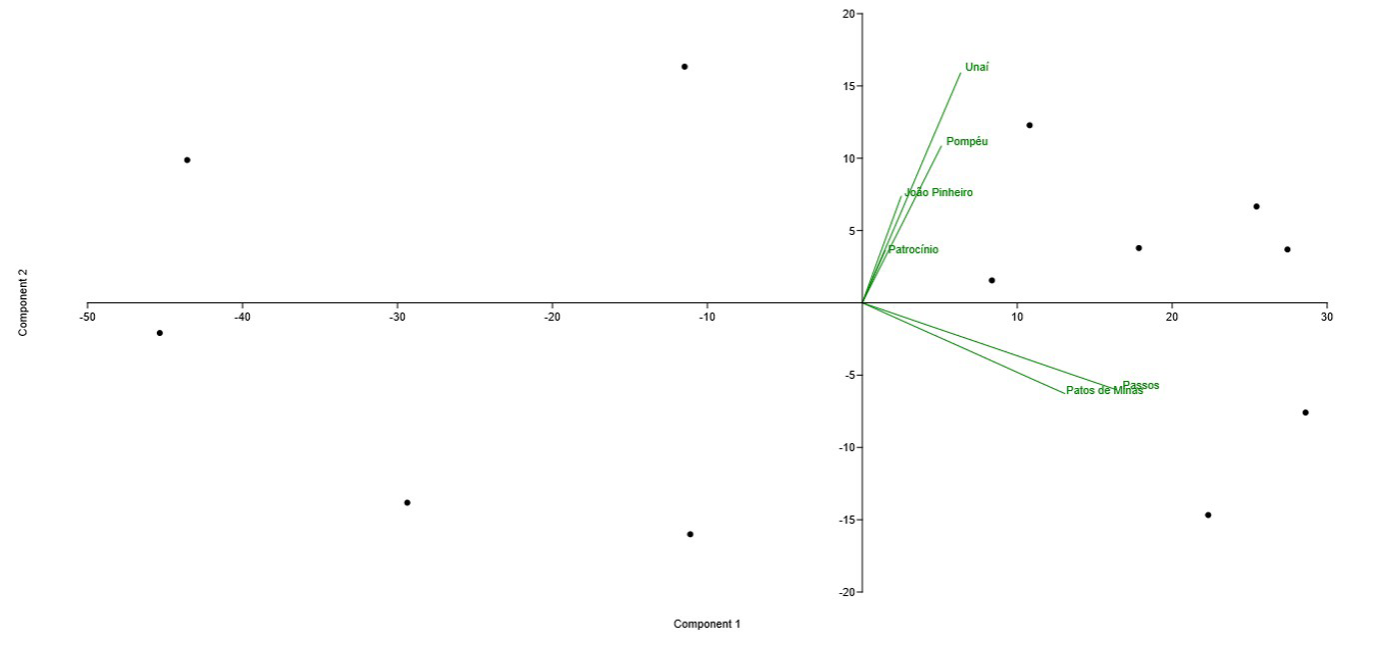
PCA results for months of the year regarding temperature highlighting the cities analyzed.

**Figure 10:**
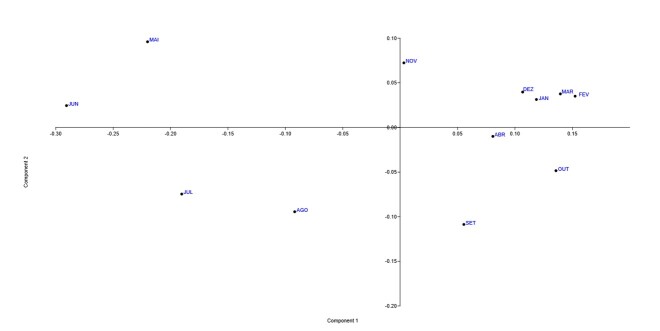
PCA results for precipitation regarding average daily volume.

**Figure 11:**
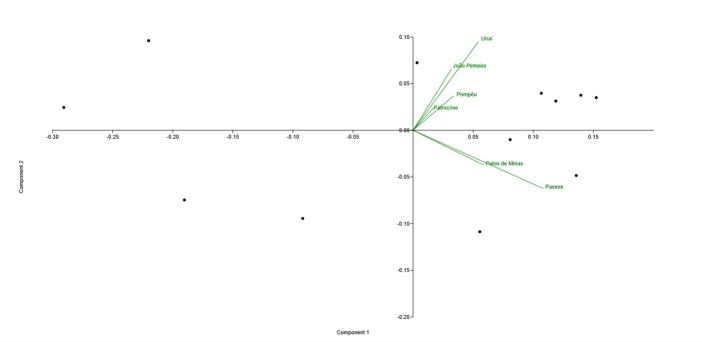
PCA results for precipitation regarding average daily volume, highlighting the analyzed cities.

**Figure 12:**
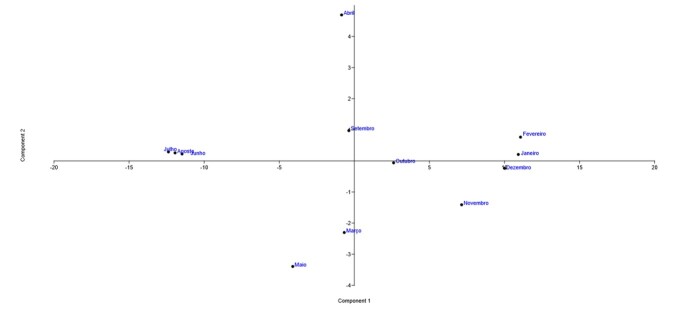
PCA results for precipitation regarding total monthly precipitation.

**Figure 13:**
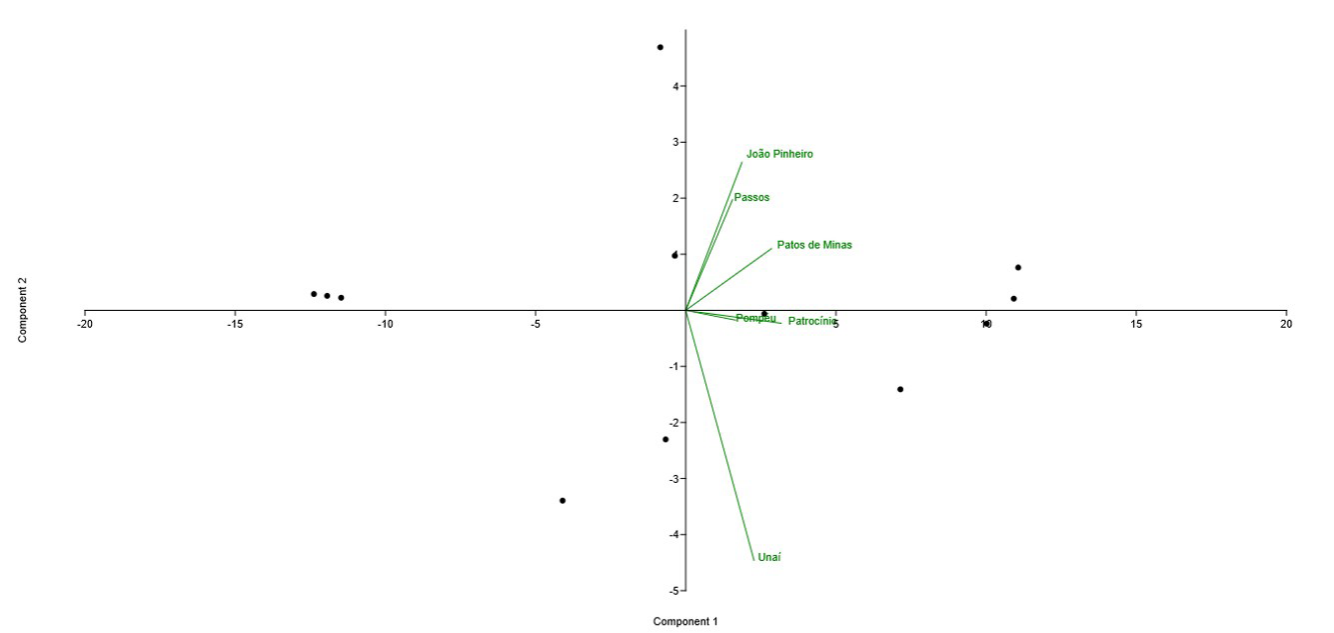
PCA results for precipitation regarding total monthly precipitation, highlighting the cities analyzed.

Principal component analyses allowed identifying similarity patterns among cities regarding temperature and precipitation. Regarding temperature, the first four months, and December, showed similar average values. The cities of Patos de Minas and Passos showed patterns that are different from the other cities, with the months of September and October with similar averages (Figures 8 and 9). Precipitation was analyzed concerning average daily volume (Figures 10 and 11) and total monthly precipitation (Figures 12 and 13). Considering average daily volume, the first quarter and the months of November and December showed similar averages, and the cities of Patos de Minas and Passos showed patterns that are different from the other cities, with April, September and October showing similar average values (Figures 9A and 9B). However, considering total monthly precipitation, January and February showed similar averages, what was also observed for June, July and August, when there was drought. The city of Unaí showed the most divergent pattern from the other cities (Figures 12 and 13).

**Figure 14:**
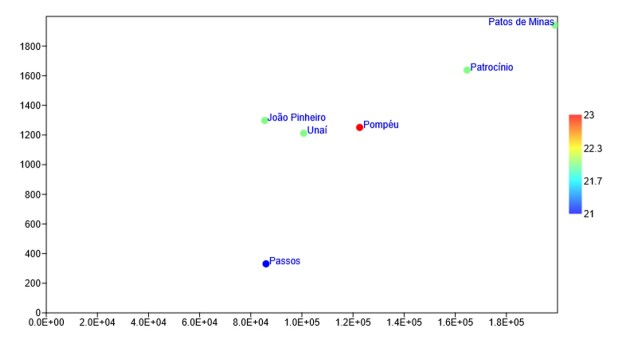
Three-dimensional relationship between annual precipitation, average temperature and milk production in municipalities of Minas Gerais. Vertical axis: total precipitation; Horizontal axis: milk production. Colored points are related to temperature values (right side scale).

In an integrated analysis of multivariate relationships (Figure 14), we confirmed the trend of increased production with increased precipitation, without wide variations in annual average temperatures, except for Passos and Pompéu. The cities of João Pinheiro, Unaí, Patrocínio and Patos de Minas presented similar temperature averages, despite the differences in milk production.

## Discussion

This study evaluated the climate-milk production relationship in MG, highlighting the importance of climate adaptation measures for sustainable production. To the best of our knowledge, this is the first study to conduct a statistically supported investigation of milk production using official data for top milk producing cities in MG. From a total of 853 cities of the state, only 12 (1.4%) had high productivity. In these cities, intensive farming system has been adopted instead of extensive pasture-based farming. The intensive system is supported by precision farming technologies that allow a more effective control of home temperature, food availability, reproductive performance and animal health (Cole & Silva, 2016; Ferrazza & Castellan, 2021). Thus, milk production is expected to be higher in this system if properly managed, considering zootechnical parameters (Almeida et al., 2021; Collard & McCormick, 2021).

The city of Pompéu stood out for its largest herd (>40.000 milked cows) and high precipitation level (1.250,8 mm/year). The city of Passos had the lowest precipitation volume (330.58 mm/year), while the city of Patos de Minas presented the highest total precipitation (1.940,6 mm/year), albeit with irregular distribution throughout the year. The city of Patos de Minas also had the highest production index among the six cities analyzed (Figure 14), all of which exhibited average annual temperatures ranging from 21 to 23°C. Statistically, precipitation alone had a greater influence on milk production than did temperature alone.

In cities in the northern and eastern regions of the state, where annual averages can exceed 30 °C, milk production was limited to 8.000 L/year (Figure 1). The thermal comfort zones for Holstein and Girolando cows are estimated to be 21 °C and 18 °C, respectively (Sammad et al., 2020; Steeneveld et al., 2024). Thus, technically, raising these breeds in the studied cities requires fewer adaptations to ensure thermal comfort compared with other regions of the state. Some cities in the Central and Triângulo Mineiro regions also exhibited higher average temperatures than those analyzed. This helps to explain the nonlinear correlation between milk production, temperature and precipitation.

Although several cities had records of high-productivity animals (Figure 5), only 12 had the highest milk production tier, most of which had a low number of animals per km^2^ (Figures 3 and 4). Interestingly, a recent survey indicated that 36 of the 100 largest milk-producing farms in Brazil are in MG, being the state with the highest concentration of dairy agribusiness in this group (MilkPoint Ventures, 2024). Some of these high-production farms can be found in the cities of Passos, Patos de Minas, Tiros, Coromandel, Patrocínio, Rio Paranaíba, João Pinheiro, and Pompéu, what partially explains the official metrics concerning total milked cows/Km^2^ and average milk volume/cow (Figures 4 and 5).

Large-scale producers typically maintain genetically selected animals that favor high milk yields. Conversely, in low-production cities, it is common for farmers to keep a small number of cows yielding ≥4.000 L/year to compete on morphological parameters and milk production standards (Gomes et al., 2018). To improve the genetic features of the animals, some cities in Minas Gerais are benefiting from the*"Mais Genética"*(“More Genetics” – in literal translation to english) government program, which provides selected semen for fixed-time artificial insemination, with technical assistance from institutions like EMBRAPA (Minas Gerais, 2022). However, due to the high cost of acquiring and maintaining genetically superior animals, these are not the majority of herds in most farming properties.

Climatic variations directly affect the metabolism and physiology of dairy cattle, primarily through thermal stress. High temperatures combined with elevated humidity compromise the thermoregulation of animals, forcing them to rely on compensatory mechanisms, such as increased respiratory rate, sweating, and peripheral vasodilation (Bayssa et al., 2021; Dias-Souza et al., 2025). Although necessary for survival, these responses redirect energy and physiological resources, resulting in hormonal imbalances, reduced feed intake and decreased milk production (Demski et al., 2019; Dias-Souza et al., 2025; Oliveira et al., 2025). This results in endocrine dysfunction, impaired ruminal activity and decreased immune function, and an increased susceptibility to infectious diseases. Milk production and quality are also affected by changes in protein and lipid synthesis, which compromise zootechnical performance (Bohlouli et al., 2021; Kolling et al., 2022; Lee et al., 2023). Variations in temperature correlate negatively with milk yield, especially in metabolically demanding breeds such as Holstein and Girolando (Barnabé et al., 2015; Habimana et al., 2024; Vilela et al., 2013).

Experimental trials with dairy cows exposed to controlled thermal environments have shown that even short-term heat stress can reduce milk production daily, especially in facilities lacking adequate shade and ventilation (Oliveira et al., 2025). Cumulative thermal stress can increase vulnerability to mastitis and metabolic disorders, highlighting the need for climate change mitigation strategies (Dias-Souza et al., 2025; Giannone et al., 2023). Cooling systems, genetic selection for heat tolerance, and climate forecasting tools are essential for minimizing climate-related losses (Osei-Amponsah et al., 2020). In intensive systems, real-time thermal monitoring allows management adjustments to reduce performance losses, such as nutritional and hormonal supplementation.

## Conclusion

The interplay between climatic parameters and physiological responses in dairy systems required a multidimensional approach. Here we provide evidence that precipitation is the dominant climatic driver of milk production in high-productivity cities in MG. These cities have achieved superior yields despite climatic challenges, whereas regions with temperatures exceeding 30°C showed limited milk production. Sustaining productivity under climate change conditions requires adaptive infrastructure, resilient genetics, and adequate animal care, to avoid health issues and thus, impact milk production. More studies are necessary to investigate the interplay of climatic changes and other relevant parameters such as nutrition and hormonal supplementation, to identify potential correlations and thus, more effective strategies to increase milk production in healthy animals.
